# Puerperal Fever

**Published:** 1884-06

**Authors:** R. L. Banta


### BUFFALO

### Medical and Surgical Journal.

Vol. XXIII. JUNE, 1884. No. 11.

### Original Communications.

### Puerperal Fever.

By Dr. R. L. Banta.

With considerable hesitation have I taken up a subject which has filled our medical journals of late, and which has been a theme of discussion in each of our late meetings.

The grand intellectual tournament which took place only a short time ago in the New York Academy of Medicine between those giants of the medical world, Thomas and Barker, with their several followers, was of more importance to humanity than twenty meetings of the "Cloth of Gold," which history has so carefully handed down to us. Lances were shivered in the encounter, but the "collision brought forth sparks," which it is to be hoped cleared up many of the dark points which surround the child-bearing woman.

Wishing to believe, with Dr. Thomas, that puerperal fever, whether it assumes a form of phlebitis cellulitis, lymphangitis or peritonitis is a puerperal septicæmia due always to an absorption of poison into the blood of the parturient woman through some solution of continuity in the tissues of the genital tract, or, in the words of Lusk, "Puerperal fever is an infectious disease, due, as a rule, to the septic inoculation of the wounds which result from the separation of the decidua and the passage of the child through the genital canal."

For then, certainly, thorough cleanliness and antiseptic precautions should produce results which would make puerperal fever a disease of the past.

Surely, then, a physician could attend a case of scarlatina, typhus, erysipelas, septicæmia or puerperal fever, and, after taking a bath, sponge his body with a saturated solution of boracic acid, change his clothes, and, after soaking his hands for a few minutes in a solution of bichloride of mercury, one part to the thousand, feel perfectly safe that he himself was not the bearer of any septic material. Then, to make things doubly sure, all the surroundings should have a thorough cleansing, and at the conclusion of labor the slightest wound of the vulva should be treated as a dangerous point, ready to receive an infectious poison.

And for fear that a micro-organism might, in its wanderings, have gained entrance into the genital canal, a solution of the bichloride, "one part to two thousand, should be injected into the vagina, and followed by a five-grain suppository of iodoform."

Many of the above precautions it has been my custom to take, especially as regards my own person, while attending an obstetrical case, for the last few years. And wishing and hoping that Dr. Thomas is right in the propositions and conclusions laid down in his paper, still, I must say, although it is my belief that most of the diseases following parturition are due to the absorption of poison through lesions of the genital tract, I cannot help but think that there is another disease, peculiar to itself.

A zymotic, infectious disease, the exact nature of which is unknown, prevails at certain periods and in certain places, sometimes, seemingly, following in the track of the obstetrician, and which no precaution seems to obviate, unless it be the removal of the physician from his practice for a time.

Such a disease it was my misfortune to meet with, in my practice, four years ago. In the short space of six weeks I met with seven puerperal fever cases, two ending fatally.

A five per cent. solution of carbolic acid (I did not, at that time, know of the virtues of corrosive sublimate as an antiseptic) was used faithfully, with a nail-brush, before each examination. Antiseptic baths, daily; new clothes from top to bottom; very few examinations by the bed-side. But still, one case followed another, not consecutively, but sometimes skipping over two or three, until, in desperation, I had my arm done up for a pretended colles fracture, by Dr. S. S. Greene and Dr. James Sloan then of this city, wishing to have an excuse to give up my obstetrical but retain my general practice. Three weeks elapsed. I was called to attend a woman in labor, who had been under anti-syphilitic treatment for the previous six months. She had had seven dead born children, and I had been watching the case with considerable interest.

Dr. Dorland was called on to help me, as some fear was expressed that I would not be able, under the circumstances, to manage the case. Up to the last, I was resolved not to make a vaginal examination, but as the Doctor was some time in arriving, and, as three weeks had gone by since my last case, I made up my mind to risk it, hoping, if there was any poison about my person, it had, by this time, passed away. The labor was a tedious one, but after a time, for good and sufficient reasons, the woman was delivered, with the forceps, of a living child, Dr. Dorland being kind enough to compliment me on the skillful way I had managed the case with a broken arm. (The Doctor, to this day, is not aware of the bogus fracture.)

Within twenty-four hours I was called to another confinement and within four days both women were sick with puerperal fever. My arm suddenly grew worse, and just as soon as possible I left Buffalo for the East and remained away five weeks.

While in a little town of New Hampshire, an incident occurred which made quite an impression on my mind at that time. While telling some of my friends the cause of my leaving Buffalo, they mentioned a doctor of that town who had, about ten years previous, very bad luck with his women, so much so that he was obliged to quit practice, and was then running a farm.

Perhaps the bad cases which it was my misfortune to see may have been coincidences. Perhaps one came from a particle of retained placenta or membranes, another from a neglected, lacerated perineum, or perhaps, a germ of one of the zymotic diseases was carried by myself or nurse.

But, no consideration could make me continue my obstetrical practice if four or five puerperal fever cases should follow, one shortly after another.

Now, turning the page, a more pleasant picture, at least for me, it is my wish to present before you. Until within about four months ago, covering a period of about two years, over three hundred confinement cases were under my supervision.

In spite of neglected, lacerated perineums, in spite of dirt and want of care, with the usual number of forceps and turning deliveries, and one case of turning especially (Dr. Walter Greene of this city being the consulting physician), in which the vaginal cul-de-sac was so badly torn that one hand could be passed into the abdomen back of the uterus, meeting the other hand on the outside, as in bi-manual palpation, seemingly nothing intervening but the skin; in spite of a want of precaution so much talked of, of late, unless the precaution of keeping my own person and instruments perfectly clean and using neither vaginal nor uterine injections only after manual deliveries, with the exception of one fatal case of embolism of the pulmonary artery, no one case has caused me an hour's uneasiness; nor can I recall a case in which the thermometer remained higher than 101° F. for forty-eight hours.

Four months ago one of my patients died with puerperal peritonitis; another, since our last meeting, with puerperal septicæmia. The history and treatment of this last case, with criticisms on my own want of success, will be given as the plan usually followed by me in treating this most fearful disease.

Mrs. F., primipara, 28 years of age, was delivered, March 22d, after a short labor, of a healthy child. Nothing occurred during labor worthy of remark, unless it were that a domestic trouble was worrying the mind of the patient. A slight laceration of the perineum was noticed after the birth of the placenta. It was a question whether or not this should be closed by suture, and the decision was not to do so, as the tear seemed to be of no moment.

Perhaps that was the first mistake. Nothing unusual happened unless it was a want of sleep, which was forced by chloral, until the third day, when, on making my morning call, I found my patient slightly delirious, with a temperature of 102° F., and a pulse of 110. Thinking and hoping it was the coming of the milk, the tr. of aconite and tablespoonful doses of liq. amon. acetatis was ordered to be given every hour. That was the second and grand mistake, as future events proved conclusively to my mind. On making my next visit, some twelve hours afterwards, found patient more delirious, with pulse of 130 and temperature of 104° F., with that peculiar, anxious and yellowish cast of countenance so characteristic of blood poisoning in the puerperal woman.

No chill had preceded as a warning, nor was there any cessation of the lochia, but every symptom pointed to a septicæmia of a severe type, and the cause became plainly evident when using an intra-uterine injection of the bichloride, one part to two thousand, a piece of the membranes, six or seven inches long, was washed from the womb. The injections were continued every three hours; quinine and salicylate of sodium were given to reduce temperature; chloral and hypodermic injections of morphia (there was no pain) to induce sleep. But in vain.

Twelve hours had been lost, and although the first cause had been removed, and the womb thoroughly washed, the poison had gained entrance into the general system and performed its deadly work, the patient dying on the eighth day from the hour of delivery.

As I look back now, I can easily point out three mistakes: Firstly, not removing the membranes entire, which, it seems to me, might happen to any one, and it does happen occasionally that part of the membranes and offensive pieces of the after-birth will remain some time after delivery without doing the slightest harm.

Why in one case such frightful results ensue, and in another not raise the pulse one beat, is about as easy to answer as to tell why one simple wound heals by first intention and another is followed by erysipelas. Secondly, in not closing the laceration of the perineum.

If at any time in my life I have taken the stand (and I have done so in one of our local societies) that nature would take care of most of the torn perineums (and many the one I have neglected with impunity) I have, at this present time, come to see the folly of my ways, and now I would go as far the other way and say that he who neglects to rectify, immediately, the ruptured perineum should be prosecuted for mal-practice. So easily done, and bringing, generally, such immediate good effects, saying nothing of the remote symptoms.

One stitch, so well described by Dr. Allway, of Toronto, is usually all that is necessary, with no trouble to the physician and no pain to the patient; but, on the contrary, almost immediate relief to that burning, smarting pain so often complained of, after labor, and which is generally produced by a tear of the perineum.

Thirdly, not using intra-uterine injections until twelve hours after the rise of temperature and quickening of the pulse.

This mistake, possibly, is not so easily to be avoided, for how often does the secretion of milk, or any other little disturbance, bring up the thermometer or quicken the pulse, which subsides in twenty-four or forty-eight hours, under appropriate or without any treatment.

Of course, in any case, the result showed there was a grand mistake, and, without doubt, had the cause been attacked twelve hours sooner, my patient would be living to-day; and I would say, here, intra-uterine injections, repeated every three hours for forty-eight hours without a reduction in the temperature and improvement in the other symptoms, does more harm than good. The "victorious army has gained entrance into the general system, and our washings simply bring away the rear guard."

On the other hand, if there be the slightest improvement, usually, by continuing the injections, success will be likely to be certain.

In conclusion, let me say that if I have not given the treatment of puerperal fever as a disease peculiar to itself, it is because I think the treatment which conquers the baneful effects produced by absorption of a poison through the injured genital tract will be just as likely, as far as it is known, to be successful in treating that disease, which undoubtedly prevails as an epidemic some years more than others, and which depends on some unknown cause, called puerperal fever.

